# Major Cellular and Physiological Impacts of Ocean Acidification on a Reef Building Coral

**DOI:** 10.1371/journal.pone.0034659

**Published:** 2012-04-11

**Authors:** Paulina Kaniewska, Paul R. Campbell, David I. Kline, Mauricio Rodriguez-Lanetty, David J. Miller, Sophie Dove, Ove Hoegh-Guldberg

**Affiliations:** 1 School of Biological Sciences, The University of Queensland, St Lucia, Queensland, Australia; 2 Agri-Science Queensland, Department of Employment, Economic Development and Innovation, Dutton Park, Queensland, Australia; 3 Department of Biology, University of Louisiana at Lafayette, Lafayette, Louisiana, United States of America; 4 ARC Centre of Excellence for Coral Reef Studies and Coral Genomics Group, School of Pharmacy and Molecular Sciences, James Cook University, Townsville, Queensland, Australia; 5 Global Change Institute, The University of Queensland, St Lucia, Queensland, Australia; University of California, Merced, United States of America

## Abstract

As atmospheric levels of CO_2_ increase, reef-building corals are under greater stress from both increased sea surface temperatures and declining sea water pH. To date, most studies have focused on either coral bleaching due to warming oceans or declining calcification due to decreasing oceanic carbonate ion concentrations. Here, through the use of physiology measurements and cDNA microarrays, we show that changes in pH and ocean chemistry consistent with two scenarios put forward by the Intergovernmental Panel on Climate Change (IPCC) drive major changes in gene expression, respiration, photosynthesis and symbiosis of the coral, *Acropora millepora*, before affects on biomineralisation are apparent at the phenotype level. Under high CO_2_ conditions corals at the phenotype level lost over half their *Symbiodinium* populations, and had a decrease in both photosynthesis and respiration. Changes in gene expression were consistent with metabolic suppression, an increase in oxidative stress, apoptosis and symbiont loss. Other expression patterns demonstrate upregulation of membrane transporters, as well as the regulation of genes involved in membrane cytoskeletal interactions and cytoskeletal remodeling. These widespread changes in gene expression emphasize the need to expand future studies of ocean acidification to include a wider spectrum of cellular processes, many of which may occur before impacts on calcification.

## Introduction

Coral reefs are highly productive and biologically diverse ecosystems despite the oligotrophic waters that surround them [1]. They are important to millions of coastal dwelling people across the planet, underpinning industries such as fishing and tourism [2]. Coral reefs appear to be facing a significant increase in local and global stressors [1], [3]. Global warming and ocean acidification have recently emerged as key threats to the long-term survival of coral reefs. Rapidly warming oceans are driving an increase in the frequency and intensity of mass bleaching events [3], while steadily acidifying oceans have caused large decreases in the concentration of carbonate ions and potentially the ability of marine calcifiers to precipitate calcium carbonate [4].

High levels of atmospheric CO_2_ ([CO_2_]_atm_) and subsequent ocean acidification have been implied as a major factor in several extinction events on coral reefs in geological time [5]. The ocean uptake of [CO_2_]_atm_ produces carbonic acid (HCO_3_^−^) as the carbon dioxide reacts with water. Protons (H^+^), which are formed due to the resulting dissociation of carbonic acid to bicarbonate ions (CO_3_^2−^), react with carbonate ions, forming more HCO_3_^−^ and thus reducing carbonate ions available for marine organisms [6]. This decrease in [CO_3_^2−^] leads to a reduction in the saturation state of calcium carbonate forms such as aragonite, calcite and high magnesium calcite and thus a reduction in calcification by marine organisms [4], [7]. To date, most studies of ocean acidification have focused on its impact on calcification rates [4], as opposed to targeting the physiological processes that lead to the biological deposition of calcium carbonate in these organisms and/or sustain organism health (fitness). It is now clear that overall the predicted reduction in ocean pH and [CO_3_^2−^] can be correlated with a decrease in calcification for a diverse range of marine calcifiers, however the response is variable, often non linear and there are inter and intra specific differences [4], [8], [9]. In addition, for studies conducted in the field, ocean acidification effects can be compounded by ocean warming [10]. Calcification is clearly important, but many other physiological processes may be affected in marine organisms [11], [12], [13]. By assessing these impacts we can commence unraveling cellular and physiological processes that eventually lead to a decrease in calcification rates. This in turn can provide information to explain currently observed discrepancies in calcification rates, which is important if we are to understand the full ramifications of rapid ocean acidification for coral reefs. Here, we investigate what physiological processes in *Acropora millepora* are affected by changes in ocean pH, both at the level of the phenotype and gene expression level and show that exposure to high CO_2_ drive major changes in gene expression, respiration, photosynthesis and symbiosis for the reef building coral.

## Results and Discussion

**Figure 1:**
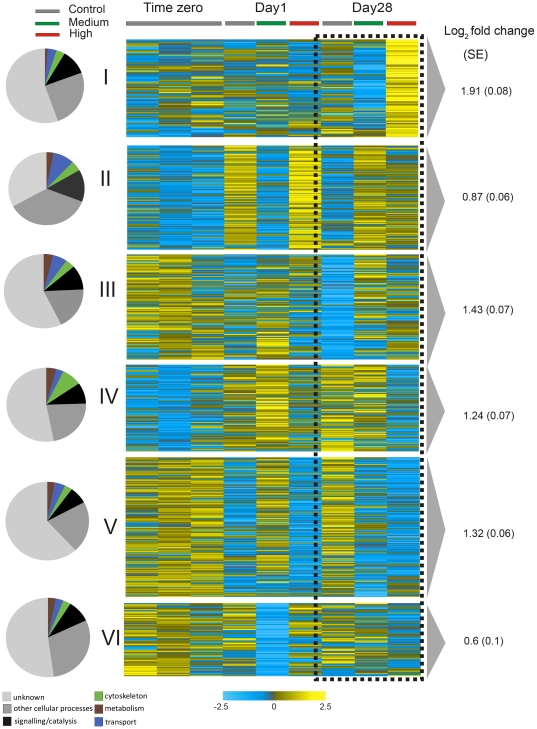
Graphical representation of differentially expressed genes in *Acropora millepora* across pCO_2_ treatments (control, medium and high) at day 1 and 28. K-means clustering was applied to group genes (synexpression clusters I–VI) by common temporal expression patterns. Yellow represents upregulation and blue represents downregulation, scale bar is on a log_2_ ratio. Each row corresponds to a transcript and each column represents the mean expression (n = 3). For each cluster average log_2_ fold changes (±SE) at day 28 are indicated and pie charts classify genes into major biological processes according to enriched GO categories.

**Figure 2:**
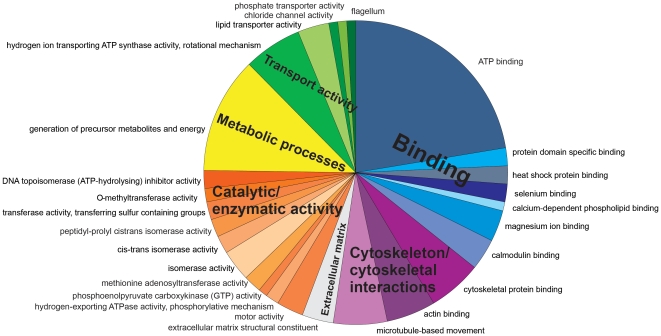
Classification analysis for *Acropora millepora* transcripts that were differentially expressed across pCO_2_ treatments (control, medium and high) at day 1 and 28. Gene enrichments (P<0.05) across GO categories are shown. The program GOEAST was used to test for enriched GO categories among differentially expressed genes. Color scheme indicates parent categories (binding, actin cytoskeleton, catalytic activity, metabolic processes and transporter activity) and individual pie segments are annotated for more specific GO categories. The sizes of the pie segments are proportional to the total number of genes enriched. The proportion of differentially expressed genes which were assigned to gene ontology categories was 55%.

**Figure 3:**
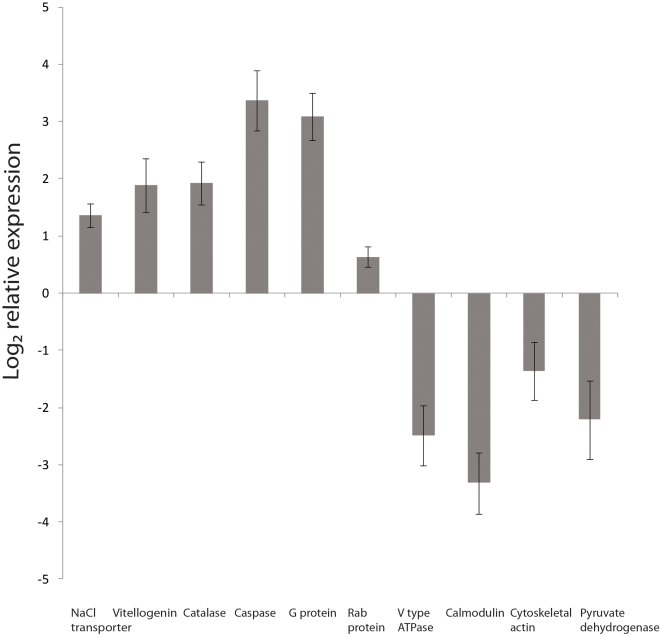
Log_2_ relative expression of selected genes using quantitative real-time PCR. Expression levels of genes are plotted as ratio of relative expression of high CO_2_ corals versus control corals at day 28. The relative expression for these selected genes was normalized to AdoHcyase and Rbl7. Bars represent standard error of the mean (n = 4).

In a study of 8606 unigenes from the coral *Acropora millepora* exposed to ambient, mid and high CO_2_ conditions as predicted by the IPCC (Table 1), we report that increases in dissolved CO_2_ after 1 and 28 days affected processes including: metabolism, membrane-cytoskeleton interactions, signaling, translation, transport, calcification, protein folding and apoptosis (Figure 1, Table S1). In total, acidification resulted in 643 differentially expressed transcripts (FDR, 5%); the largest number of these differentially expressed genes are up or down regulated in the high CO_2_ treatment compared to the control at day 28. This was also reflected in principal component analysis which showed that high CO_2_ corals at day 28 where separated from the other samples implying the greatest variation (Figure S1). Differentially expressed genes were subjected to K-means clustering in order to group genes with similar temporal expression patterns and we identified 6 major synexpression clusters (I–VI) (Figure 1). Transcripts with homology to known genes (352 transcripts, Blastx, E-score cutoff 10^−6^) were assigned to gene ontology (GO) categories and subjected to classification analysis to identify enriched GO groups (Figure 2). From the pie charts in Figure 1 which show what major GO categories genes in the synexpression clusters belong to, it is apparent that more changes in cytoskeleton interactions occur in cluster IV, more changes in signaling and catalysis occur in clusters I–III and large changes in transport occur in cluster II. Quantitative real-time PCR of ten representative genes supported the results, where each candidate gene in the qPCR followed the trends found in the microarray data with expression levels either increasing or decreasing in response to high CO_2_ conditions (Figure 3, Table S2, S3) compared to control corals at day 28. Changes in response to high and mid CO_2_ conditions for day 1, where less gene expression changes occurred, contained many changes in heat shock proteins and signaling which differed from changes at day 28 (Table S1). However, this study only had a single time point at a shorter time scale. It would be useful in future studies to better define changes in gene expression levels within the first few days of exposure, which would require an experiment with several time points within these first few days. It should be noted that this study used small sample sizes (n = 3 for microarray analysis and n = 4 for physiology and qPCR) and that future studies would benefit from greater sample sizes, perhaps a greater range of differentially expressed genes would be detected, and more robust conclusions drawn from the physiological data.

**Table 1:** Carbonate chemistry parameters^a^ across experimental conditions.

IPCC	pH*	ALK* (µM/kgSW)	DIC* (µM/kgSW)	(Aragonite)	pCO_2_ (matm)	CO_3_^2−^ (µmol kg^−1^)
Control (present)	8.0–8.2	2281.9±15.8	1832.4±59.4	3.93–5.21	260–440	253.8±17.9
A1B (medium)	7.8–7.9	2260.0±12.6	2165.4±51.0	1.14–3.71	600–790	145.3±33.7
A1FI (high)	7.6–7.7	2283.3±13.5	2345.5±214.4	0.77–2.85	1010–1350	89.5±13.0

* **Measured values.**

a **Parameters were calculated from measured values of pH, total alkalinity (ALK), dissolved inorganic carbon (DIC), temperature (25°C) and salinity (35 ppm), using the program CO2SYS.**

**Figure 4:**
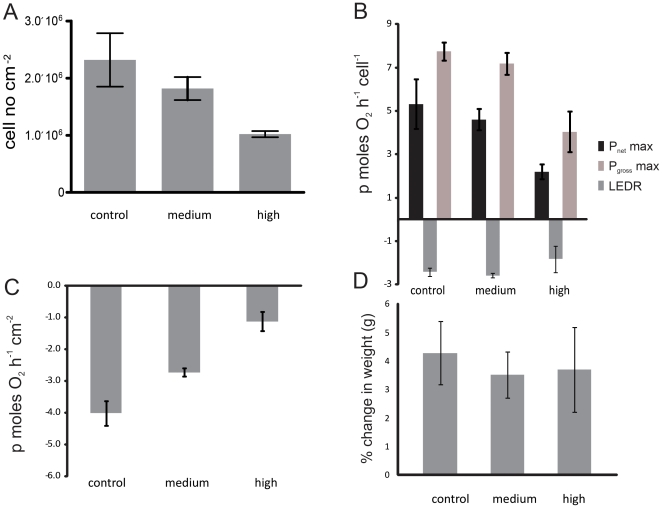
The effect of increasing CO_2_ in seawater (control, medium and high) after 28 days on coral-algal physiology. (A) *Symbiodinium* cell number in reef-building coral, *Acropora millepora* (B) photosynthetic capacity per symbiont cell measured as P_net_ max, light enhanced dark respiration (LEDR), and P_gross_ max (P_net_ max−LEDR) (C) dark respiration(R _dark_) and (D) relative calcification/growth as % change in weight (g) of coral branches over the 28 day experimental period. Error bars represent the standard error of the mean (n = 4).

Changes at the mRNA level, where the majority of differentially expressed genes were found at day 28 in the high CO_2_ treatment, were confirmed by physiological measurements (Fig. 4). *Acropora millepora* branches lost *Symbiodinium* cells in response to changes in ocean chemistry, (Figure 4A; Kruskal Wallis test, H _2,12_ = 7.54,p = 0.023), where after a 28 day exposure, *Symbiodinium* populations in the high CO_2_ treatment were reduced (1.02×10^6^±5.34×10^4^) to less than half the density compared to control branches (2.3×10^6^±4.68×10^5^) (Figure 4A). The remaining symbiont cells also became less productive and the photosynthetic capacity (as measured by P_net_ max cell^−1^ and P_gross_ max cell^−1^) was reduced. There was a 60% reduction (Kruskal Wallis test, H _2,12_ = 8.34, p = 0.015) in P_net_ max cell^−1^ and a 50% reduction (Kruskal Wallis test, H _2,12_ = 7.73, p = 0.021) in P_gross_ max cell^−1^ (P_net_ max−LEDR) in the high CO_2_ treatment compared to the control (Figure 4B). Decreasing rates of gross photosynthesis per *Symbiodinium* cell, compounded by reduced *Symbiodinium* populations, may lead to a reduction in photoassimilates translocated to the host coral. These changes are likely to have long-term negative effects on host growth and fecundity, with the prospect of increased susceptibility to disease and mortality, especially if *Symbiodinium* populations fail to recover rapidly [14]. The observed decrease in P_gross_ max is consistent with previous acidification studies [11], [15]; however, in our study there was no change in LEDR per remnant *Symbiodinium* cell among CO_2_ conditions (Kruskal Wallis test, H _2,12_ = 1.65, p = 0.437). This may be due to the application of very different light conditions to Crawley et al [15] which exposed coral to sub-saturation light intensities and only had a short experimental time scale. More importantly, there was a 3-fold downturn in dark respiration per coral surface area (Figure 4C), (Kruskal-Wallis test, H _2, 12_ = 6.71, p = 0.035), which is typically associated with a decline in host maintenance and/or growth [16]. Rapid growth, either as tissue growth or calcium carbonate deposition necessitates high respiration rates, but the observed reductions in the rate of respiration can suggest suppression of growth rates and/or metabolism. Physiological changes in this study preceded any observable changes in calcification/growth as determined by changes in buoyant weight, as there was no difference in branch calcification/growth rates between the 3 treatments after the 28 day incubation (Kruskal Wallis test, H _2,12_ = 0.50, p = 0.778) (Figure 4D), despite the downturn in both energy production and respiration observed in the high CO_2_ treatment. This result may reflect that in this case, observable effects on calcification/growth rates require longer experimental incubation, as the buoyant weight technique may be too insensitive to measure the potential small changes in calcification/growth that may have occurred.

### Metabolism

Changes to metabolic rates are a common outcome of environmental stress [17]. Changes in gene expression suggest that *Acropora millepora* may have reduced its metabolism under high CO_2_ conditions at day 28 (Figure 1 cluster IV–VI, Figure 2, Table S1), mirroring the oxygen flux change (Figure 4). There was an overall down-regulation of genes involved in the tricarboxylic acid (TCA) cycle and the mitochondrial electron transport chain (Table S1), indicating reduced oxidative metabolism and capacity to generate ATP and NADPH. There was also an upregulation of triglyceride lipase and Acyl-CoA dehydrogenase (Figure 1 cluster I, II, Table S1), which may point to an increase in the breakdown of lipids for energy use [18], [19]. Interestingly, there was an increase in mitochondrial transcripts for ATPase (Figure 1 cluster II, Table S1). Cellular apoptosis is often preceded by an increase in mitochondrial ATPase activity, resulting in an influx of potassium, the activation of caspases and ultimately cell death [20]. Metabolic suppression has been shown in a range of marine organisms in response to CO_2_ fluctuations [13], [21]. The majority of energy needs in tropical reef building corals are supplied by the photosynthetic endosymbionts [22], but host heterotrophy can occasionally meet host requirements [23]. Depressions in aerobic metabolic activity due to mitochondrial disruptions can undermine the viability of host cells regardless of the trophic source of organic carbon supplied into the TCA cycle. In this particular case, metabolic suppression due to acidosis is likely to have long-term fitness costs.

### Acid-Base Regulation and Ion/Macromolecule Transport

Maintaining pH homeostasis is critical to a range of cellular functions [24]. Studies of acid-base regulation and hypercapnia suggest significant physiological challenges for marine fish and worms [13], [25]. There are cases where mitochondrial energy production is tied to acid-base regulation through HCO_3_^−^ transport [26], bi-direction H^+^ pumping by F_0_F_1_ ATPase [20], or Na^+^/H^+^ and Cl^−^/HCO_3_^−^ transporters on the cell membrane [25]. Membrane proteins play an integral role in: pH homeostasis of the cell, membrane lipid composition and cell shape maintenance [27]. For *A. millepora*, 28 days of high CO_2_ conditions resulted in changes in membrane transporters (Figure 1, 2, Table S1). Specifically, there was downregulation of proton channels (V-type proton ATPases), phosphate transport and protein transport at the cell membrane (Figure 1 cluster IV,V, Table S1). At the same time, sodium and potassium transporters, cell membrane receptors and an ABC transporter were upregulated (Figure 1 cluster I, Table S1). In eukaryotes, ABC type transporters are involved in the export of unwanted molecules, such as toxins [28] from the cell. V- type proton ATPases at the cell membrane serve to acidify the extracellular environment which in turn activates a series of signaling cascades [29]. In the cnidarian ectoderm, plasma membrane proton ATPase activity has been tied to CO_2_ uptake [30]. A decrease in this transporter may indicate a decrease in CO_2_ uptake under acidification stress. Due to concurrent increases in energy saving ion gradient transporters such as Na^+^/H^+^ exchangers, the decrease in V- type ATPases for proton transport may also be the result of an active suppression of the more costly ATP dependent ion transporters [31]. In addition, a lipid transporter was upregulated in the high CO_2_ treated corals at day 28 (Figure 1 cluster II, Table S1), a change not found in acid base regulation of other marine organisms [13], [21], [25], perhaps implying changes to the lipid configuration of the cell membrane as a response to ocean acidification [32].

### Stress Response Mechanisms

Abiotic changes are likely to elicit a cellular stress response (CSR), a universally conserved mechanism to protect macromolecules within cells from the potential damage that physical, chemical or biological stressors may cause. The CSR can increase the tolerance temporarily to the stressor, and remove already damaged cells through apoptosis [33]. Transcripts of *A. millepora* that encode a number of cellular defenses, and transcripts involved in maintenance of protein integrity (molecular chaperones) were downregulated (Figure 1 cluster V, Table S1), whilst genes, protecting the cells against oxidative stress through oxidoreductase activity (eg. Catalase, FAD linked oxidase and selenoprotein [34], [35], [36]) and involved in apoptosis (caspase 3, TRAF3, p53 inducible protein 11 and programmed cell death protein 4 [37], [38], [39]), were upregulated in high CO_2_ treated corals at day 28 (Figure 1 cluster I, II, III, Table S1). Bcl-2, MALT1 and API-5, potential inhibitors of apoptosis [40], [41], [42] were downregulated (Figure 1 cluster V, Table S1). The upregulation of apoptotic transcripts is consistent with the upregulation of mitochondrial ATPase described above, which together point to disruption in the mitochondrion leading to cell death [20]. An increase in apoptosis may reflect that prolonged environmental stress, and either a lack of cell pH homeostasis or elevated maintenance costs, has resulted in cell damage. The loss of *Symbiodinium* cells and an increase in transcripts alleviating oxidative stress may point to impairment in the photosynthetic apparatus in the dinoflagellate symbiont or an impairment of the coral mitochondria [14]. This, in turn, would increase the presence of oxygen radicals in the host tissues and imply cell damage potential. The fact that high CO_2_ conditions resulted in overall downregulation of protein folding transcripts, may be a sign that the coral tissue may no longer have the capacity to maintain these integral services. Interestingly, at day one of the high CO_2_ treatment there was an upregulation of Heat shock protein 40, a change not found at Day 28 (Figure 1 cluster II, Table S1). It must be noted however, that the two other main heat shock proteins (hsp) were not differentially expressed between treatments (hsp 70 and 90) but were maintained at a high expression levels, and their presence may be sufficient for the integrity of newly made proteins. Calnexin and alpha mannosidase transcripts were upregulated (Figure 1 cluster II, Table S1) which would increase the quality control and protein folding ability in the endoplastic reticulum for proteins that will then be further transported to the golgi complex [43], [44]. It is possible that the other downregulated chaperones could be temporarily reduced while awaiting more favorable environmental conditions. A coral c-type lectin, which is involved in innate immunity in corals [45] was downregulated (Figure 1 cluster V, Table S1) under high CO_2_ conditions, indicating that this cell stress response may not be responding appropriately, and this decrease may compromise the coral further as in a stressed holobiont, susceptibility to pathogens may increase [46].

### Ca^2+^ Ion Binding/Transport and Cell Communication

Several calcium (Ca^+^) ion binding proteins were downregulated in high CO_2_ treatments at day 28 (Figure 1 cluster IV, V, Table S1). Transcripts for calcium-binding receptors that are potentially involved in innate immunity [47], [48] were also suppressed, implying an adverse change in signaling potential at the cell membranes. Downregulation of calmodulin, FKBP12 and EGF-hand proteins also implies potential disruption in cell calcium homeostasis [49], [50], [51], as these calcium binding proteins control the Ca^2+^ release from ryanodine receptors (RyR) within the endoplasmic reticulum (ER), which is an intracellular Ca^2+^ storing organelle [49], [50], [51], [52]. Calpain, an important Ca_2_^+^ activated protease that has roles in membrane-cytoskeleton interactions, signal transduction, cell differentiation and apoptosis [53], was upregulated. Changes in these calcium binding proteins indicate that certain signaling pathways may have been altered.

### Membrane-Cytoskeleton Interactions

Exposure to high seawater CO_2_ concentrations for 28 days resulted in several differentially expressed genes involved in membrane-cytoskeleton interactions and cytoskeletal remodeling (Figure 1, 2, Table S1). It is possible that the change in regulation of these transcripts reflects a change in proteins involved in cytoskeletal interactions, cytoskeletal organization, intracellular transport, cell shape integrity and cell motility [54], [55] [55]. Specifically, there was downregulation of cytoskeletal actin 1, centractin, radixin and coatomer epsilon subunit and radixin (Figure 1 cluster IV, V, Table S1), whilst there was upregulation of tubilin and Lgl tumor suppressor unit (Figure 1 cluster I, II, Table S1). The actin cytoskeleton is important in a diverse range of processes such as cell motility, contractibility, mitosis and cytokinesis, intracellular transport, endocytosis and secretion. In addition, it has been suggested that actin is also involved in regulation of gene transcription through changes in the cytoskeletal actin dynamics or assembly of transcriptional regulatory complexes [55]. Actin is also an important part of the nuclear complex being required for the transcription of RNA polymerases and is also involved in the export of RNAs and proteins from the nucleus [55]. It is possible that the downregulation of cytoskeletal actin in high CO_2_ conditions reflects a change in the regulation of gene transcription of proteins involved in cytoskeletal interactions. In addition this downregulation can imply changes in the intracellular transport, plasma membrane interactions and cell shape/integrity. There was also an upregulation of alpha tubilin, which forms a constituent of the microtubule filaments, involved in cytoskeletal organization and vesicle transport. Downregulation of coatomer epsilon subunit implies changes in protein trafficking between the endoplasmic reticulum and the Golgi complex, while upregulation of Lgl tumor suppressor unit indicates changes to events controlling cell polarity [56], [57]. Cell volume control changes have been recorded in other marine organisms such as crabs in response to hypercapnia [58], and similar changes may be occurring in the stressed coral cells. The downregulation of Radixin, an important protein involved in linking the plasma membrane to the cytoskeleton using actin rich surfaces [59], supports the downregulation of cytoskeletal actin. Centractin, or Actin Related Protein 1 (ARP1), was also downregulated under higher CO_2_ stress, and this is an important activator of cytoplasmic vesicle movement [60]. In contrast, at day one in high CO_2_ stressed corals, there was an upregulation of Radixin and Centractin, or Actin Related Protein 1 (ARP1) (Figure 1 cluster VI, Table S1), indicating that different changes in cytoskeletal interactions were occurring at this stage. The cytoskeleton has profound effects on the plasma membrane. At times, there may be uninhibited lateral diffusion of lipids and proteins across the plasma membrane; the influx of these molecules can be regulated by the membrane-cytoskeleton links. These become obstacles to free diffusion through diffusion-limited lipid domains [54]. It may be that changes in these membrane-cytoskeleton links in this study reflect changes in transport across the membrane.

### Rab/Ras GTPases

Exposure to increased CO_2_ concentrations for 28 days lead to an up (Figure 1 cluster II, Table S1) and downregulation (Figure 1 cluster V, Table S1) of transcripts that resemble members of the Rab/Ras GTPases families (small G-proteins). The Ras GTPase superfamily is a small monomeric group of GTPases, which are involved in cell proliferation and cell signaling events in response to external stimuli. Disruption of the Ras signaling pathway is a key component in the progression of tumor growth [61]. The Rab GTPase family is part of the Ras GTPase superfamily and plays a key role in many membrane-trafficking events in eukaryotic cells, such as exocytosis. This group of proteins which tightly associates with the cell membrane is involved in transport vesicle formation, actin and tubilin motility, docking and membrane fusion. Rab proteins are active when bound to GTP and are inactive when bound to GDP [62], [63]. In its active state, the Rab protein regulates the transport of lipids and proteins between distinct membrane bound organelles through interactions with downstream effector proteins which are recruited onto the membranes [63]. Small G-proteins are implicated in most cellular events where plasma membrane-cytoskeleton interactions or plasma membrane shape changes (plasma membrane deformations) occur. The observed upregulation in members of these small monmeric GTPases most likely reflects changes in the cell membrane and cytoskeletal interactions to accommodate changes in external seawater chemistry.

### Extracellular matrix

Changes in the extracellular matrix (ECM) have previously been implied to potentially affect calcification [34], [36]. Our expression patterns indicate that only two transcripts encoding previously described ECM proteins changed after 28 days under mid CO_2_ exposure. SEC13L1 was upregulated (Figure 1 cluster III, Table S1) while peroxidasin was downregulated (Figure 1 cluster IV, Table S1). At day 28 under high CO_2_ exposure, there was also a downregulation to a predicted protein in the extracellular matrix (Table S1). This implies that small changes to calcification may have started occurring and that perhaps with a longer experimental incubation time more ECM and calcification related transcripts would have been differentially expressed. This overall supports our findings at the phenotype level where no change in calcification/growth was found (Figure 4). Overall in this study there were fewer changes in transcripts which may be involved in calcification, in response to ocean acidification, compared to gene expression studies with corals exposed to thermal stress where changes to the following transcripts were observed; collagen α-1, ECM matrix metalloprotease, papilin, carboxypetidase inhibitor SmC1, procollagen, galaxin and SCP-like extracellular protein [34], [36].

### Cell-wide Responses by Corals to Ocean Acidification: A Model

**Figure 5:**
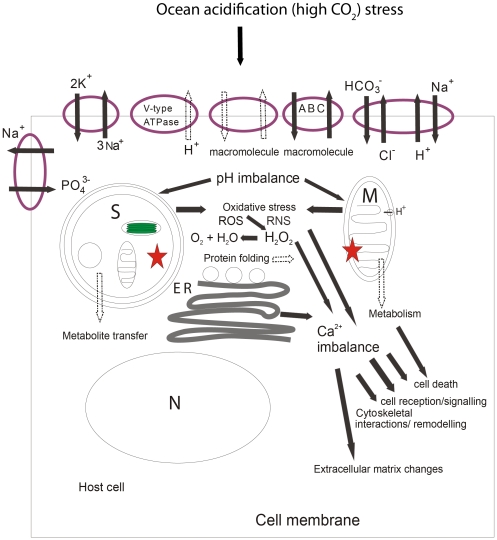
A proposed model of cellular events occurring as a result of ocean acidification. These changes lead to compromised health in *Acropora millepora* (reduction in symbiont cells and decreased photosynthesis and respiration). The schematic depicts an endodermal cell which contains the symbiont cell. Cellular events depicted here are likely to also occur in other cell types which do not contain symbionts, especially the acid base changes at the cell membrane. Changes in carbonate chemistry lead to changes in acid base regulation and cell membrane transporters. Acid base regulation may not be sufficient leading to acidosis within the cell. This could increase reactive oxygen species (ROS) due to a disruption (⋆) in the *Symbiodinium* cell (S) and/or in the coral host mitochondrion (M), which may also produce reactive nitrogen species (RNS). The overall oxidative stress and a disruption to calcium stores at the endoplasmic reticulum (ER) can lead to calcium imbalance. This in turn leads to events such as changes in the extracellular matrix, cytoskeletal remodeling, changes in cytoskeletal interactions, disruption to cell reception and signaling potential, and an increase in cell death. Moreover disruption in both the host mitochondrion and *Symbiodinium* cell leads to a decrease in metabolism and a decrease in metabolite transfer from the symbiont cell. In addition the disruption in the host mitochondrion can also lead to cell death. For the cell membrane transporters black arrows indicate upregulation and white arrows indicate downregulation.

To highlight the differences between acidosis which may be a factor in this study from the impact of hypercapnia seen in other marine organisms, we purpose a model (Figure 5) of cell-wide, coral host response to high CO_2_ stress. This model attempts to account for the classic acidosis response (acid-base regulation and metabolic depression) and the novel responses observed in the studied coral (apoptosis, signaling events, calcium homeostasis, cytoskeletal remodeling, cytoskeletal-membrane interactions and oxidative stress). The coral specific responses may result from increased reactive oxygen species (ROS) and/or increased reactive nitrogen species (RNS) created from a disturbance in the *Symbiodinium* cell, the host mitochondria, or both [14], [34]. Upregulation of catalase, FAD-linked oxidase and selenoprotein indicates that there may be an increased amount of ROS in the coral cells [64], [65]. Increased ROS/RNS can result in a disruption to the calcium homeostasis [34]. The role of internal [Ca^2+^] increase in coral bleaching has been suggested previously [34]. The downregulation of calmodulin (CaM), FKBP12 and EF-hand proteins under high CO_2_ stress indicates that there may be a disruption to the Ca^2+^ homeostasis [49], [50], [51]. Modifications of the actin cytoskeleton, membrane-cytoskeleton interactions and cell receptor/adhesion properties will be affected by a disruption in Ca^2+^ homeostasis and metabolic suppression [34], [66]. Both oxidative stress and an increase in intracellular Ca^2+^ can lead to apoptosis and changes in transcripts indicate that both the NF-kB and p53 apoptotic pathways [67], [68] were upregulated. Changes in predicted proteins in the extracellular matrix may imply changes in or restructuring of the extracellular matrix. Our model suggests that similar cellular events are occurring under acidosis induced bleaching to those reported for thermally induced bleaching [14], [34], [36], but with the addition of changes to acid-base regulation and mitochondrial ATPase activity.

On our present greenhouse trajectory, we are likely to use all the >4000 Gt of carbon present in the global fossil fuel reserves by 2400. This will significantly acidify the oceans for thousands of years [69] and take them to a point not seen in tens of millions of years [70]. Our study highlights the imperative to investigate the impacts of ocean acidification on processes other than those involved in biomineralisation. Also, there is a need for more studies investigating the effects of naturally occurring changes in pCO_2_ on marine calcifiers *in situ*. This is a priority, if we are to understand the fate of the many supporting roles that corals contribute to the maintenance of coral reefs.

## Materials and Methods

### Experimental Design

A total of 20 branches (7–8 cm long) were collected from 4 healthy colonies of the reef building coral *Acropora millepora* on Heron Island Reef flat (23 33′S, 151 54′E), Great Barrier Reef, Australia. Coral branches were affixed onto cut 15 mL falcon tubes using Selleys Knead It Aqua (Padstow, Australia) and Selleys autofix super glue (Padstow, Australia). The affixed branches were then placed onto a rack which was deployed back to the Heron Island reef flat, where they remained for 4 weeks, exposed to natural light and flow regimes in order to recover from handling. Following this acclimation period they were transferred to aquaria with running seawater and under ambient light (with shade cloth see below) and ambient temperature (26°C) conditions for 10 days. For each treatment there were four randomly distributed aquaria and for each *A. millepora* colony, branches were evenly distributed across treatments, with 6 branches per colony in each of the treatment tanks (ambient, mid and high – see below). Branches were designated to be used either for respirometry assays and physiology measurements or microarray analysis. The experiment was run for 28 days and coral branches were sampled, snap frozen in liquid nitrogen and stored at −80°C for later analysis at time zero, day 1 and day 28. For each time point, two branches per colony were sampled and one branch was used for physiology and one branch was used for genomic analysis.

The experimental set up consisted of 12 (4 aquaria per treatment) flow-through aquaria (80 L) under natural light and a layer of shade cloth which resulted in photosynthetically active radiation levels of a maximum of 859.6 µmole quanta m^−2^ s^−1^ and a daily average level of 433.6±8.6 µmole quanta m^−2^ s^−1^ for the light period of the day. Aquaria were supplied with unfiltered seawater which was being pumped straight from the reef flat on Heron Island into CO_2_ mixing tanks, and then distributed across aquaria. The control aquaria were receiving seawater from the Heron Island reef flat where the natural diurnal variability in pH ranged between 8.0–8.2 due to tidal changes and metabolic activity on the reef flat, which corresponded to a pCO_2_ range of 260–440 ppm. The pCO_2_ ranges in the two acidification treatments were controlled by a CO_2_ dosing control system (Aquacontroller III, Neptune Systems, Carlsbad, CA, USA) which used pH readings in the large 300 L CO_2_ mixing tanks, to either open or close solenoid valves (Dupla Australia, Littlehampton, Australia) and would control the amount of CO_2_ being added to the mixing tanks. The medium CO_2_ treatment was controlled to a pH target of 7.8–7.9 corresponding to 600–790 ppm. The high CO_2_ treatment was targeted to a pH range of 7.6–7.7 corresponding to 1010–1350 ppm. Temperature, pH and light levels were recorded throughout the experiment and total alkalinity across control and CO_2_ treated aquaria was determined with a Mettler Toledo T50 automated titrator, with 0.1 M HCl and 130 g seawater samples using the Gran titration method in a two-stage, potentiometric, open-cell titration following the method of [71]. Acid concentrations and the alkalinity measurements were calibrated at the beginning of each run using Dickson certified reference sea water standards (Andrew Dickson, SIO, Oceanic Carbon Dioxide Quality Control). Dissolved Inorganic Carbon (DIC) was sampled into 50 mL glass vials after filtering with a 0.45 µM syringe filter, fixed with 15 µL saturated mercuric chloride and then sealed with a rubber lid and aluminum cap (Wheaton, USA). DIC samples were then run on a custom DIC system with a LICOR gas analyzer (Rob Dunbar lab, Stanford, USA) with a Dickson sea water reference run every 7 samples. Carbon species concentration and aragonite state were determined for each treatment using the CO2SYS program using the dissociation constants from [72] and refit by [73] using TA, DIC, pH, and temperature measurements [74].

### Respirometry Measurements

Coral branches designated for physiological measurements were used for respirometry assays. The coral branches were dark adapted for at least an hour before respirometry assays, which were performed after dusk. Branches were placed in 70 cm^3^ clear acrylic chambers with an inserted optode sensor connected to an Oxy4 v2 system (PreSens, Regensburg, Germany). The chambers were placed within an acrylic container (on top of a magnetic stirrer), which was connected to a water bath keeping the temperature constant throughout the assays at 26°C, the ambient water temperature at Heron Island during the course of the experiment. Chambers were filled with seawater from respective experimental treatments in the aquaria. Photosynthesis vs irradiance curves (P-E curves) were conducted by using the actinic light source of an imaging pulse amplitude modulation fluorometer (iPAM, Walz, Effeltrich, Germany), and exposing coral branches in chambers to 0, 10, 20, 55, 110, 925 µmol quanta m^−2^ s^−1^ for 10 min and then followed by exposure at 1075 and 1250 µmol quanta m^−2^ s^−1^ for 5 min each, and the run was concluded by a 10 min incubation at 0 µmol quanta m^−2^ s^−1^. Respirometry rates were normalized to cell number and coral surface area. The exposure to different light levels enabled the calculation of P-E curve parameters [75] following methods in [15]; dark respiration (R_dark_) estimated from the initial 10 min dark incubation, sub-saturation photosynthetic efficiency (α) derived from the regression line slope of the low irradiance levels (10, 20,55, 110 µmol quanta m^−2^ s^−1^) in relation to the estimated E_k_, photosynthetic capacity (P_net_ max) was estimated as the greatest rate of oxygen evolution at the high irradiance levels (925, 1075, 1250 µmol quanta m^−2^ s^−1^), and finally light-enhanced dark respiration (LEDR) was determined from the oxygen consumption in the last dark incubation post irradiance exposure.

### Population Density and Chlorophyll *a* Content of *Symbiodinium*

The cell density and pigment content of *Symbiodinium* were measured by removing tissue from coral fragments by air- brushing frozen fragments in 5 mL 0.06 M phosphate buffer (pH 6.65). The homogenate was centrifuged at 4000×g for 5 min. The supernatant was removed and the remaining dinoflagellate pellet was re-suspended in filtered seawater (0.45 µm) and separated into aliquots that were used for pigment quantification and *Symbiodinium* cell counts. *Symbiodinium* pigment quantification aliquots were centrifuged at 4000×g for 5 min, the supernatant was removed and 1 mL of 100% cold methanol was added to the pellet. The solution was sonicated on ice cold water for 10 min and then centrifuged at 4000×g for 5 min. The supernatant was collected and transferred into a tube. This process was repeated until complete pigment extraction was achieved (when the final supernatant was clear). The total final extracted solution was filtered (0.45 µm) and used for pigment separation in a Shimadzu SCL – 10 HPLC linked to a Shimadzu SPD – M10A photodiode array detector, using the column and method described in [76] with solutions A (methanol: acetonitrile: aquose pyridine, 50∶25∶25 v∶v∶v) and B1 (methanol∶ acetonitrile∶ acetone, 20∶60∶20 v∶v∶v). A standard for methanol extracted pigment chlorophyll *a* was used for quantifying pigments and normalized on a per cell basis. *Symbiodinium* cell counts were estimated using eight randomly selected replicates counted using a haemocytometer (Boeco, Germany) on a Zeiss standard microscope; the counts were normalized to coral surface area in cm^2^, as obtained by dipping coral fragments into paraffin wax following the method of [77].

### Coral Growth Rate Estimation

*Acropora millepora* branch growth/calcification rate was estimated using the buoyant weight method [78]. Coral branch weights for samples across treatments were measured at time zero, day 1 and day 28. The branches were suspended by a thin fishing line below a precision balance (Mettler Toledo) and the weight was recorded. The growth/calcification rate was calculated as a relative unit by subtracting the initial weight (g) from the final weight (g) and converting this to a percent change in weight over the course of the experimental period.

### Statistical Analysis

All data were tested for normality and homogeneity of variance and where assumptions were violated, the data were corrected by transformations. Non-parametric equivalents of tests were used in cases where assumptions were violated despite transformations. A Kruskal Wallis test was used to determine the effect of changes in CO_2_ concentrations on *Symbiodinium* density, branch calcification/growth, P_net_ max, P_gross_ max, LEDR and R_dark_. To test for significance on the expression levels of mRNAs from quantitative real time PCR between control and high CO_2_ levels, for each gene a Welch t-test was used. All statistical analyses were performed using STATISTICA 7.0 (Statsoft Inc., Tulsa, USA).

### Microarray Description

The microarrays used in this experiment were printed at the Adelaide Microarray Facility (Australia) and consisted of 18,432 spots derived from the same amount of cDNA clones, including 290 spots representing positive and negative control and representing 8606 unigene clusters [79]. These microarrays are the 3^rd^ generation cDNA microarrays designed for *Acropora millepora* [80]. The selection of clones, methodological approach for the cDNA library construction and the fabrication of microarrays are explained in [81].

### Hybridization of Arrays

Total RNA was extracted from each sample using Trizol (Invitrogen) following manufacturer's instructions. The integrity and quality of total RNA was assessed using a Bioanalyzer (Agilent Technology). Only samples showing intact RNA (RNA Integrity number >8), were used for probe construction. cDNA probe synthesis was performed from 1000 ng total RNA using Superscript Reverse Transcriptase (Invitrogen) and a 2 pmol Genisphere 900 3DNA Dendrimer from a Genisphere 3DNA-900 microarray kit according to the manufacturers' instructions. We used a reference two-colour microarray design, where, for each array, the sample was labeled with Cy5 and the reference, consisting of pooled RNA from control treatments and time zero, was labeled with Cy3. In total, 27 arrays were hybridized, as each array represented a sample from a treatment and a time point (n = 3), only 3 out of the 4 colonies were used for microarray hybridization. Microarrays were pre-hybridized and hybridized with the labeled samples using the Genisphere 3DNA-900 microarray kit following the manufacturer's instructions and using a dynamic hybridization system (MAUI, BioMicro Systems). Prior to and post hybridization, the microarray slides were washed three times (wash 1∶ 2×SSC 0.2% SDS at 65°C for 15 min, wash 2∶ 2×SSC at room temperature for 10 min, wash 3∶ 0.2×SSC at room temperature for 10 min). Slides were scanned using a GenePix ® 4200 scanner (Axon Instruments) and image acquisition was performed using the software GenePix ® Pro 5 (Molecular Devices, CA, USA).

### Microarray Analysis

Normalization and data analysis of acquired array slides was performed using R (R Development Core Team, 2008) and the limma package [82]. The details for the methodology of analyzing differential gene expression using empirical Bayes shrinkage of variance and linear regression models can be found in [83]. Normexp (75) corrected signal intensities were used, as it has been shown to be a well performing background correction method, which best stabilizes variance as a function of intensity, compared to more standard and common methods [84]. Print-tip loess normalization was applied within slides [85] while scale normalization was applied between slides, in order to ensure that distributions were similar between arrays. Both normalization procedures equalize for differing amounts of host RNA input [36]. Effectiveness of normalization procedures was verified through M (the log ratio of the spot fluorescence intensity) vs A (the log of the average spot fluorescence intensity) plots. Minimal or no fluorescence was observed for probes which contained salmon sperm DNA and primers, and should not hybridize, while controls which were expected to hybridize showed a range of fluorescence intensities. Differentially expressed genes were identified based on an assumed false discovery rate of 5% and sequence-wise p-values were adjusted through the Benjamini and Hochberg method [86]. Sets of contrast lists of differentially expressed genes between control, medium and high CO_2_ treatments at day 1 and day 28 were created (643 transcripts) (Table S1, Additional file 1), in addition contrast lists were created between groups of samples at t0 so that any potential differences due to “tank effects” could be subtracted from subsequent analysis; in total 3 genes were subtracted from subsequent contrast lists. Differentially expressed genes at day 1 and 28 were then assembled into 6 different clusters based on their temporal gene expression patterns, using K-means clustering analysis in the TIGR TMEV software [87], assuming that genes with similar cellular pathways share common temporal expression patterns. In addition Principal component analysis was also carried out in the TIGR TMEV software [87]. Differentially expressed genes which had homology to known genes (352 transcripts, Blastx, E-score cutoff 10^−6^), were assigned to GO categories and subjected to classification analysis using the hypogeometric test and a false discovery rate of 5% in GOEAST [88] to identify enriched GO groups. Microarray data has been deposited in the Gene Expression Omnibus Database (GSE28697).

### Validation by Quantitative PCR

Expression patterns of candidate genes from each functional group of coral genes differentially expressed in response to increased CO_2_ treatment (sodium and chloride transporter, V type ATPase, vitellogenin, catalase, caspase 3, calmodulin, cytoskeletal actin, pyruvate dehydrogenase, G protein and rab protein) were validated through quantitative Polymerase Chain Reaction (qPCR). Total RNA (1000 ng) was reverse transcribed with a Superscript Vilo cDNA synthesis kit (Invitrogen) following manufacturer's instructions. Specific primers amplifying approximately 100–200 bp PCR products were designed for the genes (Table S4) chosen to be validated from the microarray data. Transcript levels were determined by qPCR using the Corbett Rotor Gene 6000 thermal cycling system (Qiagen), following the manufacturer's instructions (Qiagen, CA, USA) and PCR conditions (95°C for 10 min, followed by 40 cycles of 95°C for 15 sec and 60°C for 1 min). Triplicate first strand diluted 1∶10 cDNA aliquots (1 µL) from each sample were used in 20 µL PCR reactions with 2 µM primers and a SYBR Green PCR master mix (Warrington, Cheshire, UK). For each candidate gene control versus high CO_2_ samples (4 replicates) at day 28 were tested, as this was the conditions under which major physiological changes occurred, and the greatest differential gene expression was present. A no template control as well as a no reverse transcription control was performed for each gene and treatment to ensure that the cDNA samples did not have DNA contamination. In addition to ensure that gene expression data was representing coral host genes, primer specificity of all primers was tested through PCR using the qPCR primers and cDNA and genomic DNA from *Symbiodinium* sp. as a template to ensure no amplification. The comparative delta CT method was used to determine relative quantities of mRNA transcripts from each sample. Each value was normalized to two reference genes adenosyl-homocysteinase (AdoHcyase) and ribosomal protein L7 (Rpl7). The selection of reference genes for this experiment was done by using a pool of reference genes (Table S4) and analyzing the expression stability using the GeNorm software [89]. For this study the most stable expression was found for adenosyl-homocysteinase (AdoHcyase) and ribosomal protein L7 (Rpl7) (M value = 0.253) and a minimum of two reference genes was recommended (V2/3 = 0.126). Relative expression values were for each gene were calculated by showing a ratio of treatment relative expression over control relative expression on a log_2_ scale, which provides a similar appearance of up and down regulation [41].

## Supporting Information

Figure S1 **Principal component analysis of gene expression for 3 acidification treatments; control (c), medium (m) and high (h) and 3 time points: T0 (t), day 1 (d) and day 28 (m).**
(EPS)

Table S1 **Annotated list of differentially expressed transcripts for** ***Acropora millepora*** **between CO_2_ at day 1 and day 28 (from Additional file 1), as determined by empirical Bayes moderated statistics.** Transcripts are assembled into 6 different clusters according to K-means clustering and based on their temporal gene expression patterns. Annotations represent blastx, results with an E-score cutoff 10^−6^.
(XLS)

Table S2 **Summary of t-test with Welch's correction results for candidate transcripts validating microarray results for control and high treatments at day 28 (n = 4).**
(EPS)

Table S3 **Comparison of mean log2 fold changes for candidate transcripts from qPCR and microarray data analysis, at day 28 for high versus control corals.** The correlation coefficient (R) is 0.93.
(EPS)

Table S4 **List of candidate genes used in qPCR expression analyses.**
(EPS)
